# Implementation of a shared decision-making training program for clinicians based on the major depressive disorder guidelines in Japan: A multi-center cluster randomized trial

**DOI:** 10.3389/fpsyt.2022.967750

**Published:** 2022-08-12

**Authors:** Yoshikazu Takaesu, Yumi Aoki, Yui Tomo, Takashi Tsuboi, Miho Ishii, Yayoi Imamura, Hisateru Tachimori, Koichiro Watanabe

**Affiliations:** ^1^Department of Neuropsychiatry, Graduate School of Medicine, University of the Ryukyus, Okinawa, Japan; ^2^Department of Neuropsychiatry, Kyorin University School of Medicine, Tokyo, Japan; ^3^Psychiatric and Mental Health Nursing, St. Luke’s International University, Tokyo, Japan; ^4^Department of Clinical Data Science, National Center of Neurology and Psychiatry Hospital, Tokyo, Japan; ^5^Senzoku Stress Coping Support Office, Tokyo, Japan; ^6^Endowed Course for Health System Innovation, Keio University School of Medicine, Tokyo, Japan

**Keywords:** clinical guidelines, cluster randomized controlled trial, decision aid, depression, shared decision making

## Abstract

**Background:**

Although shared treatment decision-making with patients requires attention, it is not widely implemented, particularly in the field of psychiatry. The aim of this study was to assess whether a shared decision-making (SDM) training program for clinicians based on the major depressive disorder (MDD) guidelines improved the perceived involvement of the decision process for patients with MDD.

**Methods:**

A multi-center cluster-randomized controlled intervention of a clinician training program based on the Japanese MDD guidelines using related decision aids compared to usual care was conducted among 56 clinicians from 23 institutions. A total of 124 patients with MDD were enrolled in this study. The primary outcomes were the scores of the Shared Decision Making-Questionnaire-9 (SDM-Q-9) and Decision Conflict Scale (DCS) after the first visit to the outpatient clinics. The secondary outcomes were patients’ satisfaction, quality of life, trust in clinicians, and depressive symptoms. Additionally, we evaluated all the observed outcomes at the first and third months of follow-up.

**Results:**

The scores of the SDM-Q-9 in the SDM training program group were significantly higher than those in the control group at the first visit. However, no significant difference in the DCS scores was found between the two groups. There was no intervention effect for secondary outcomes and the outcomes at the first- and third-month follow-up visits.

**Conclusion:**

The clinician training program based on the Japanese MDD guidelines can be useful for implementation of SDM. Additional research is needed to confirm the efficacy of this SDM training program.

**Clinical trial registration:**

[https://www.umin.ac.jp/], identifier [UMIN000034397].

## Introduction

Major depressive disorder (MDD) is considered a common disease affecting more than 264 million people worldwide (1), and is expected to be the second leading cause of global health burden by 2030 (2). Therefore, it is important for patients with MDD to receive evidence based-treatments, based on the treatment guidelines. Owing to continuous development of literature in this area, several evidence-based guidelines for MDD have been published in several countries (3–7). These guidelines recommend shared decision-making (SDM), a process of patient-centered care, in which patients also play a role in medical decisions. For example, the World Federation of Societies of Biological Psychiatry Guidelines propose thorough discussion with patients regarding the goals, advantages, and disadvantages of long-term therapy, adequate related information, as well as patients’ personal goals (5). The MDD guidelines published by the Japanese Society of Mood Disorders also describe that patients and clinicians should collaborate in treatment decision-making while sharing the lines of evidence provided by the guideline (6).

To date, several SDM randomized controlled interventions for patients with MDD have been conducted in many countries. LeBlanc et al. conducted an SDM intervention for antidepressants in primary care using decision aid cards that describe general antidepressant considerations, such as weight change, sleep, libido, discontinuation, and cost (8). Aoki et al. developed an SDM program for first-episode mood disorders, which consisted of treatment options, presentation consultation, decision aid booklets, decision coaching by a nurse, and decision-making consultation with a clinician (9). Perestelo-Perez et al. developed a web platform decision aid for MDD, where patients can learn about symptoms, types of depression, and treatment options as a preparation for the consultation with a clinician (10). Raue et al. focused on primary care of elderly depressed minority (patients aged ≥65 years) and provision of a brief SDM intervention consisting of a meeting with a nurse followed by two weekly telephone calls (11). The aforementioned interventions suggest that SDM interventions could improve patients’ participation in decision-making, satisfaction, knowledge, and decisional conflict in depression treatment (8–11).

In contrast to increasing SDM research as described above, SDM has not been widely implemented in clinical practice (12). To ensure its wide-scale adoption in practice, SDM training for clinicians is crucial (12). However, only a few studies have examined the effects of SDM training among clinicians who treat MDD cases. The aim of this study was to assess whether an SDM training program for clinicians based on the Japanese MDD guidelines improved the perceived involvement of the decision process for patients with MDD.

## Materials and methods

### Study design

This study was designed as a multi-center, matched-pair cluster randomized controlled trial in 23 psychiatric institutions (4 general hospitals, 8 psychiatric hospitals, and 11 psychiatric outpatient clinics) addressing outpatients who newly visited the institution and were diagnosed as having MDD according to the DSM-5 criteria (13). This study was approved by the Ethics Committee of Kyorin University and conducted after obtaining written informed consent from the patients. The study protocol was registered at the University Hospital Medical Information Network registry (UMIN000034397).

### Participants

All participants fulfilling the inclusion criteria were consecutively screened for the trial at the time of their first visit to the study institutions. The inclusion criteria were as follows: first visit to the psychiatric institution, diagnosis of MDD according to the DSM-5 criteria (13), and age between 20 and 65 years. The exclusion criteria were as follows: severe MDD requiring hospitalization, suicidal ideation, severe physical disease, and diagnosis of substance abuse and dementia.

### Randomization

We randomly assigned 23 medical institutes (cluster randomization) to either the intervention (SDM group) or the control group (treatment as usual). Cluster randomization was performed in three categories (general hospitals, psychiatric hospitals, and psychiatric outpatient clinics) using computer-generated random numbers. According to the matched-pair cluster randomized design, the randomization sequences were per block of three categories using SPSS syntax, which generated random numbers (0 = control, 1 = intervention). This syntax was prepared by a data manager with no involvement in this trial (HT). The random allocation sequence was conducted by research assistants, prior to the initiation of the intervention and data collection, independent of a research team.

### Blinding

Due to cluster randomization at the institution level and the nature of the intervention, blinding of the clinicians and patients was not feasible. To reduce the risk of bias, research assistants, independent of this research team, carried out the data collection. During the inclusion process, independent research assistants were blinded to the allocation of the condition.

### Development of decision aids

Decision aids (Das) are tools to facilitate the SDM process between patients and clinicians and help in achieving a mutual decision according to the patients’ preferences (14). The authors (YA and KW) developed decision aids for MDD following the international patient decision aid standards instrument (15) and confirmed their feasibility (9, 16). In this study, we modified the previous decision aids by referring to lines of evidence described in the MDD guidelines published by the Japanese Society of Mood Disorders (6). The MDD treatment guideline explains that patients with MDD should receive supportive psychotherapy regardless of the depression severity. Apart from supportive psychotherapy as a fundamental intervention, the guideline outlines evidence-based treatments depending on the depression severity. For those with mild depression, medication treatments and/or cognitive behavioral therapy can be considered (6). In contrast, for those with moderate to severe depression, medication or/and modified electrical convulsive therapy should be provided initially, and thereafter, systematized psychotherapy should be considered as an option (6). Therefore, we developed two kinds of decision aids for patients with MDD. One was named “decision aid for mild MDD patients according to treatment guideline”; it was used for those with mild depression with two options, namely, medication treatment and systematized psychotherapy (Supplementary material 1). The other aid was named “decision aid for patients with moderate MDD according to treatment guideline”; it was used for those with moderate depression with two options, namely, medication treatment and modified electrical convulsive therapy with one extra option of systematized psychotherapy (Supplementary material 2). The first chapter of each decision aid addressed the explanation of MDD. Following this, tables, including the advantages and disadvantages of each option, were provided, which subsequently led to value clarification of each option.

### Interventions

The clinicians of the intervention group participated in a 1-day training program consisting of several sessions aimed to facilitate a better understanding of the SDM process based on the MDD treatment guidelines. The training was organized by the authors (YoT, YA, TT, and KW) who were familiar with both the MDD guidelines and the SDM process, as supervised by the author (KW) who had learned the SDM concepts and its clinical skills from Dr. Hamann, a leading SDM researcher in psychiatry (17). The training program included three morning sessions (30 min/session) addressing the MDD treatment guidelines. Then, a lecture regarding recovery and SDM was provided as a luncheon session (30 min). In the afternoon, the participants role-played SDM consultations using the decision aids described above. Each group had four to five clinicians. The role-play session included two approaches: one for acquiring general SDM skills (40 min) and the other for practicing SDM skills focusing on social functioning and recovery (50 min). Before each role-play session, a small lecture was given. Group members who participated in role-play had a discussion and provided feedback to each other (20–30 min). During role-play and discussion, the group was facilitated by the researchers (YoT, YA, TT, and KW). The timetable of the 1-day training program is provided in Supplementary material 3 and the lecture material and role-play scenarios are available upon request from the authors. The clinicians of the control group did not participate in any SDM training and treated patients with MDD as usual.

### Outcomes

#### Primary outcomes

We used two validated self-report SDM-related questionnaires as primary outcomes (double primary outcome). The first one was the Shared Decision Making-Questionnaire-9 (SDM-Q-9) (18) to assess patients’ perceived participation in decision-making. The questionnaire consisted of nine items, each describing one step of the SDM process. It was developed to determine the extent to which the patients felt involved in the process, by scoring nine items from 0 to 5 points on a 6-point Likert scale; the scale ranged from “completely disagree” (0 points) to “completely agree” (5 points). Summing up all items led to a raw total score between 0 and 45 points. Multiplication of the raw score by 20/9 provided a score transformed to range from 0 to 100 points, where 0 indicated the lowest possible level of SDM and 100 indicated the highest extent of SDM. Scores <25 points were associated with implementation of decisions, and those >37.5 points were associated with uncertainty about implementation. The other was the Decision Conflict Scale (DCS) (19), which assessed decisional conflict as uncertainty of action among several options. Each of the 16 items was scored from 0 (strongly agree) to 4 (strongly disagree). Besides a total score, the DCS included five dimensions (information, support, clarification of value, certainty, and decision quality). High scores indicated more decision conflict, which means that patients reported having received less information, support, and clarification and poor decision quality concerning decision making. To calculate the total scores of five dimensions, the item scores were summed, divided by the number of items, and multiplied by 25. Thus, the scores ranged from 0 to 100 points.

#### Secondary outcomes

We used the validated self-report questionnaire to measure the secondary outcomes: Client Satisfaction Questionnaire (CSQ-8) (20), EuroQol 5 Dimension (EQ-5D) (21), Trust in Physicians Scale (TPS) (22), and Quick Inventory of Depressive Symptomatology self-report (QIDS-SR) (23).

### Endpoint

We set the primary endpoint as after the initial visit to services. We also evaluated all outcomes at the first and third months after the first visit as follow-up secondary endpoints.

### Sample size

A sample size calculation was performed prior to the study initiation, to detect a difference between the two groups with an expected clinically relevant medium effect size on the primary outcome patients’ rated SDM-Q-9. We used a medium effect size of *d* = 0.5 according to a previous study (24) because this is considered to be a clinically meaningful effect. A sample size of 64 patients per group was needed to obtain a usual power beta = 0.80 with an intra-cluster correlation coefficient (ICC) of 0.03. We calculated that with an ICC of 0.03 and an inflation factor of 1.45 at the institution level; a sample size of 93 per group was needed [design effect = 1 + (*m* − 1) × ICC] (*m* = number of individuals in a cluster = 10). Considering a dropout rate of 20%, we calculated at least 102 patients per group to certify a sufficient power.

### Statistical analyses

The Student’s *t*-test for continuous variables and Chi-square test for categorical variables were used for the comparison of characteristics, clinical variables, and medication for MDD between the two groups.

Before the following analysis, we performed multiple imputation by chained equation (MICE) in cases of missing values. For each score, in the case of unanswered items, the items and scores were treated as missing values if ≥80% of items were answered. If <80% of the items were answered, the individual was excluded from the analysis. We used information on intervention, cluster, and other items of the scores for imputation. Imputation methods were logistic regression for the intervention variable, the predictive mean matching for cluster information and other items of the score, and linear regression for the totals of scores. One hundred complete datasets were generated. If multiple imputation was used, the estimated values of the parameters and standard errors were merged by Rubin’s rule (25).

To assess the effect of the intervention on continuous primary and secondary outcomes, mixed-effects linear regression models were performed. The models included the binary covariate that represents intervention. Random effects were introduced into the intercept term for considering heterogeneity of baseline among medical institutes. The ICCs for the outcomes were estimated using the variance components of the mixed-effects model. The effect sizes (Cohen’s *d*) were calculated by dividing the between-group difference by the pooled standard deviation (SD). The thresholds for interpreting the effect size were: small 0.00–0.32, medium 0.33–0.55, and large ≥ 0.56 (26).

The comparison of characteristics of patients was performed using SPSS version 25 for Windows (IBM Corp., Armonk, NY, United States). The MICE procedure and the following analysis were conducted with R version 3.6 (R Foundation for Statistical Computing, Vienna, Austria).

## Results

### Study flow chart

**FIGURE 1:**
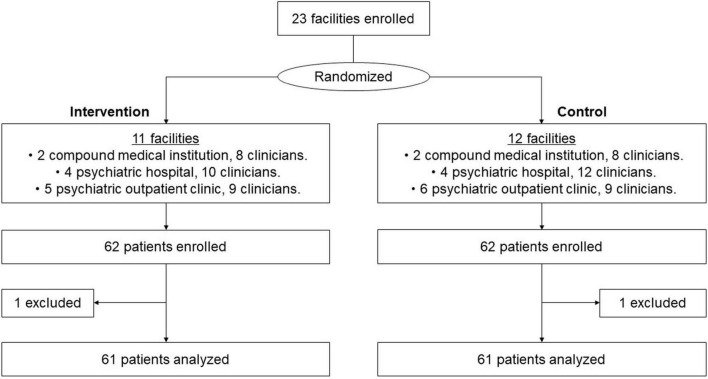
CONSORT flow chart.

Figure 1 shows the flow chart of this study. A total of 23 psychiatric medical facilities (4 general hospitals, 8 psychiatric hospitals, and 11 psychiatric outpatient clinics) with 56 clinicians were randomly assigned to the two groups. Eleven institutions with 27 clinicians were assigned to the intervention group. We held six sessions of the 1-day program from August 2018 to March 2019. All clinicians in the intervention group participated in the 1-day program. In total, 124 outpatients with MDD who met the criteria of this study were recruited between November 2018 and January 2020. Finally, the data of 61 patients with MDD in the intervention and control groups, respectively, were analyzed.

### Baseline characteristics

There were significant differences between the intervention and control groups in the rate of sex (men: 49.2 vs. 75.4%; *p* = 0.005), educational backgrounds (college graduate: 44.3 vs. 63.9%; *p* = 0.045), antipsychotic consumption (6.6 vs. 31.2%; *p* = 0.001), and benzodiazepine consumption (27.9 vs. 54.1%; *p* = 0.006). No significant difference in other variables was observed between the two groups (Table 1).

**TABLE 1:** Characteristics of study participants.

	Value (number (%) or mean ± SD)		*P*
	Intervention	Control	
Age (years)	36.3 ± 10.7	38.6 ± 11.9	0.129
Sex (male: female)	30 (49.2): 31 (50.8)	46 (75.4): 15 (24.6)	0.005
Educational background (college graduate: no)	27 (44.3): 34 (55.7)	39 (63.9): 22 (36.1)	0.045
Marital status (married: unmarried)	28 (45.9): 33 (54.1)	24 (42.1): 33 (57.9)	0.714
Employment status (unemployed: employed: housewife: student: other)	4 (6.6): 45 (73.8): 6 (9.8): 2 (3.3): 4 (6.6)	1 (1.6): 48 (78.7): 2 (3.3): 4 (6.6): 6 (9.8)	0.291
Family history of psychiatric illness (yes: no)	13 (21.3): 48 (78.7)	14 (23.0): 47 (77.0)	1.000
Living alone (yes: no)	21 (34.4): 40 (65.6)	24 (39.3): 37 (60.7)	0.708
Smoking (yes: no)	12 (20.0): 48 (80.0)	18 (29.5): 43 (70.5)	0.293
Age of onset of the disorder (years)	34.84 ± 10.13	35.73 ± 11.94	0.063
Physical comorbidities (yes: no)	11 (18.3): 49 (81.7)	16 (26.7): 44 (73.3)	0.382
**Psychotropic medication**			
SSRI (yes: no)	11 (18.0): 50 (82.0)	18 (29.5): 43 (70.5)	0.201
SNRI (yes: no)	7 (11.5): 54 (88.5)	13 (21.3): 48 (78.7)	0.221
NaSSA (yes: no)	8 (13.1): 53 (86.9)	13 (21.3): 48 (78.7)	0.338
Other antidepressants (yes: no)*	0 (0.0): 61 (100.0)	1 (1.6): 60 (98.4)	1.000
Antipsychotic (yes: no)	4 (6.6): 57 (93.4)	19 (31.2): 42 (68.8)	0.001
Benzodiazepines (yes: no)	17 (27.9): 44 (72.1)	33 (54.1): 28 (45.9)	0.006

Data presented as percentages of category or means ± SDs.

SSRI, selective serotonin reuptake inhibitor; SNRI, serotonin noradrenaline reuptake inhibitors; NaSSA, noradrenergic and specific serotonergic antidepressant.

*Other antidepressants: trazodone or sulpiride.

### Primary outcomes

The SDM-Q-9 score after the first visit in the intervention group [38.7 ± 0.8, 95% confidence interval (CI): 37.1–40.3, ICC = −0.007) was significantly higher than that in the control group (33.7 ± 1.0, 95% CI: 31.6–35.7, ICC = 0.363; Regression coefficients = 5.5 ± 2.1, 95% CI: 1.3–9.8, Intercept = 3.2 ± 1.6, 95% CI: 30.1–36.3, *d* = 0.688, *p* = 0.012).

There was no significant difference in the DCS total score after the first visit between the intervention and control groups (31.3 ± 2.0, 95% CI: 27.4–35.2, ICC = −0.085 vs. 36.1 ± 1.7, 95% CI: 32.7–39.5, ICC = −0.047; Regression coefficients = −4.8 ± 2.6, 95% CI: −10.1 to 0.4; Intercept = 36.1 ± 1.9, 95% CI: 32.4–39.8, *d* = 0.331, *p* = 0.072). In the sub-item scores of DCS, the score of “informed” (intervention vs. control, 24.7 ± 2.0, 95% CI: 20.8–28.6, ICC = −0.044 vs. 34.2 ± 2.6, 95% CI: 28.6–39.2, ICC = −0.076; Regression coefficients = −9.6 ± 3.2, 95% CI: −15.9 to 3.3, Intercept = 34.4 ± 2.3, 95% CI: 30.0–38.9, *d* = 0.546, *p* = 0.003) and “value” (intervention vs. control, 27.8 ± 2.3, 95% CI: 23.3–32.2, ICC = −0.127 vs. 39.1 ± 2.6, 95% CI: 34.0–44.2, ICC = 0.038; Regression coefficients = −11.2 ± 3.4, 95% CI: −17.9 to −4.4; Intercept = 39.3 ± 2.4, 95% CI: 34.5–44.1, *d* = 0.596, *p* = 0.001) in the intervention group after the first visit were significantly lower than those in the control group. However, no significant differences in “support” (intervention vs. control, 26.9 ± 2.3, 95% CI: 22.5–31.4, ICC = −0.085 vs. 30.1 ± 1.9, 95% CI: 26.4–33.9; Regression coefficients = −3.2 ± 3.0, 95% CI: −9.1 to 2.7; Intercept = 30.1 ± 2.1, 95% CI: 25.9–34.3, *d* = 0.196, *p* = 0.285), “uncertain” (intervention vs. control, 42.2 ± 3.1, 95% CI: 36.2–48.2, ICC = −0.049 vs. 43.1 ± 2.4, 95% CI: 38.4–47.8, ICC = −0.098; Regression coefficients = −1.1 ± 3.9, 95% CI: −8.8–6.5; Intercept = 43.5 ± 2.7, 95% CI: 38.1–48.9, *d* = 0.053, *p* = 0.772), and “effective decision” (intervention vs. control, 33.4 ± 2.1, 95% CI: 29.2–37.6, ICC = −0.029 vs. 33.9 ± 1.7, 95% CI: 30.6–37.1, ICC = −0.034; Regression coefficients = −0.5 ± 2.7, 95% CI: −5.9 to 4.9; Intercept = 33.9 ± 1.9, 95% CI: 30.0–37.7, *d* = 0.031, *p* = 0.865) were found between the two groups (Table 2).

**TABLE 2:** Comparison of primary outcomes after first visits.

	Intervention		Control		Regression coefficients (SE)	95% CI	Intercept (SE)	*d*	*P*
	Mean (SE)	ICC	Mean (SE)	ICC					
SDM-Q-9	38.7 (0.8)	−0.007	33.7 (1.0)	0.363	5.5 (2.1)	1.3 to 9.8	33.2 (1.6)	0.688	0.012
**DCS**									
Total	31.3 (2.0)	−0.085	36.1 (1.7)	−0.047	−4.8 (2.6)	−10.1 to 0.4	36.1 (1.9)	0.331	0.072
Informed	24.7 (2.0)	−0.044	34.2 (2.6)	−0.076	−9.6 (3.2)	−15.9 to 3.3	34.4 (2.3)	0.546	0.003
Values	27.8 (2.3)	−0.127	39.1 (2.6)	0.038	−11.2 (3.4)	−17.9 to −4.4	39.3 (2.4)	0.596	0.001
Support	26.9 (2.3)	−0.085	30.1 (1.9)	0.006	−3.2 (3.0)	−9.1 to 2.7	30.1 (2.1)	0.196	0.285
Uncertain	42.2 (3.1)	−0.049	43.1 (2.4)	−0.098	−1.1 (3.9)	−8.8 to 6.5	43.5 (2.7)	0.053	0.772
Effective decision	33.4 (2.1)	−0.029	33.9 (1.7)	−0.034	−0.5 (2.7)	−5.9 to 4.9	33.9 (1.9)	0.031	0.865

SE, standard error; ICC, intra-class correlation coefficients; 95% CI, 95% confidence interval; DCS, Decision Conflict Scale; SDM-Q-9, Shared Decision Making-Questionnaire-9.

### Secondary outcomes

There were no significant differences in CSQ-8, EQ-5D, TPS, and QIDS-SR scores after the first visit between the two groups (Table 3).

**TABLE 3:** Comparison of secondary outcomes after first visits.

	Intervention		Control		Regression coefficients (SE)	95% CI	Intercept (SE)	*P*
	Mean (SE)	ICC	Mean (SE)	ICC				
CSQ8J	25.6 (0.4)	−0.042	25.8 (0.4)	0.185	−0.2 (0.8)	−1.7 to 1.3	25.8 (0.5)	0.779
EQ-5D	0.57 (0.04)	0.240	0.60 (0.03)	0.021	−0.1 (0.1)	−0.2 to 0.1	0.6 (0.1)	0.385
TPS	36.4 (0.4)	0.186	36.0 (0.4)	0.128	0.6 (0.7)	−0.8 to 2.0	35.8 (0.5)	0.387
QIDS-J	14.9 (0.6)	−0.025	13.6 (0.6)	0.033	1.3 (0.9)	−0.5 to 3.1	13.6 (0.6)	0.155

SE, standard error; ICC, intra-class correlation coefficients; 95% CI, 95% confidence interval; CSQ-8-J, Client Satisfaction Questionnaire; EQ-5D, EuroQol 5 Dimension; TPS, Trust in Physician Scale; QIDS-J, Quick Inventory of Depressive Symptomatology.

Concerning follow-up evaluations, there were no significant differences in DCS, SDM-Q-9, CSQ-8, EQ-5D, TPS, and QIDS-SR scores at the first- and third-month follow-up visits between the two groups (Table 4).

**TABLE 4:** Comparison of outcomes at the time of first and third month follow-ups.

		SDM		Control		Regression coefficients (SE)	95% CI	Intercept (SE)	*P*
		Mean (SE)	ICC	Mean (SE)	ICC				
SDM-Q-9	1 month	36.7 (0.9)	0.247	33.8 (1.3)	0.302	3.0 (2.6)	−2.5 to 8.4	33.3 (1.9)	0.263
	3 months	37.1 (0.9)	0.351	36.0 (0.9)	0.339	1.8 (2.5)	−3.2 to 6.7	34.7 (1.8)	0.485
**DCS**									
Total	1 month	29.2 (1.9)	0.242	35.0 (2.4)	0.121	−6.4 (4.2)	−15.5 to 2.7	35.6 (3.0)	0.151
	3 months	28.9 (2.5)	0.145	28.3 (2.2)	0.100	0.5 (4.0)	−8.8 to 9.7	29.1 (2.9)	0.912
Informed	1 month	27.3 (2.4)	0.353	33.5 (2.9)	0.251	−6.4 (6.2)	−19.7 to 6.8	34.5 (4.5)	0.315
	3 months	26.7 (2.4)	0.121	26.1 (2.6)	0.081	0.7 (3.9)	−9.1 to 10.4	26.4 (2.7)	0.873
Values	1 month	29.4 (2.2)	0.295	37.4 (3.0)	0.170	−9.0 (5.5)	−20.8 to 2.9	37.9 (3.9)	0.126
	3 months	30.8 (3.0)	0.118	29.8 (2.7)	0.012	0.9 (4.2)	−10.0 to 11.7	30.0 (2.9)	0.846
Support	1 month	23.3 (2.4)	0.248	27.8 (2.5)	0.082	−4.8 (4.2)	−14.3 to 4.8	28.1 (2.9)	0.285
	3 months	23.7 (2.4)	0.210	24.4 (2.1)	0.155	−0.7 (4.3)	−10.4 to 9.1	25.5 (3.1)	0.880
Uncertain	1 month	36.7 (2.6)	−0.229	42.6 (2.9)	−0.044	−5.9 (3.9)	−13.7 to 1.9	42.6 (2.7)	0.136
	3 months	34.8 (3.4)	−0.024	35.2 (3.2)	−0.043	−0.4 (4.7)	−9.6 to 8.9	35.2 (3.2)	0.939
Effective decision	1 month	29.1 (2.1)	0.172	33.8 (2.7)	0.094	−5.3 (4.3)	−14.7 to 4.0	34.3 (3.1)	0.240
	3 months	28.7 (2.9)	0.093	26.5 (2.0)	0.173	2.0 (4.5)	−7.7 to 11.8	27.6 (3.2)	0.659
CSQ8J	1 month	25.8 (0.4)	0.322	25.6 (0.6)	0.233	−0.2 (0.8)	−1.7 to 1.3	25.8 (0.5)	0.694
	3 months	26.0 (0.5)	0.447	26.3 (0.5)	0.200	−0.2 (1.1)	−2.7 to 2.3	25.9 (0.8)	0.852
EQ-5D	1 month	0.70 (0.04)	−0.021	0.72 (0.03)	0.014	−0.0 (0.1)	−0.1 to 0.1	0.7 (0.0)	0.734
	3 months	0.79 (0.03)	−0.085	0.81 (0.02)	−0.113	−0.0 (0.1)	−0.1 to 0.1	0.8 (0.0)	0.635
TPS	1 month	36.7 (0.5)	−0.193	35.9 (0.4)	0.143	0.6 (0.7)	−0.8 to 2.0	35.8 (0.5)	0.301
	3 months	36.8 (0.7)	−0.182	36.7 (0.5)	0.070	0.0 (0.8)	−1.8 to 1.9	36.7 (0.6)	0.960
QIDS-J	1 month	10.7 (0.7)	−0.105	9.4 (1.2)	0.188	1.4 (3.5)	−5.6 to 8.5	9.2 (3.0)	0.684
	3 months	9.1 (0.9)	−0.155	7.8 (0.6)	−0.063	1.2 (1.4)	−1.6 to 4.1	7.8 (0.9)	0.379

SE, standard error; ICC, intra-class correlation coefficients; 95% CI, 95% confidence interval; DCS, Decision Conflict Scale; SDM-Q-9, Shared Decision Making-Questionnaire-9; CSQ-8-J, Client Satisfaction Questionnaire; EQ-5D, EuroQol 5 Dimension; TPS, Trust in Physician Scale; QIDS-J, Quick Inventory of Depressive Symptomatology.

## Discussion

This is the first cluster randomized-controlled study to assess the effects of SDM training program for clinicians based on the Japanese MDD guidelines. We found that the SDM-Q-9 score of patients was significantly higher than that in the control group after the intervention. In contrast, there were no differences in the DCS score between the two groups. There were also no differences in secondary and long-term outcomes between the two groups. Accordingly, further research is needed to confirm the efficacy of this program.

Although the SDM-Q-9 score was significantly higher in the patients than in the control after the intervention, no significant difference in the DCS score was found between the two groups after the intervention. The discrepancy might be caused by differences in the nature of the two SDM questionnaires. The SDM-Q-9 assesses patient perceptions of SDM. For example, the items include SDM-related questions, such as “my doctor made it clear that a decision had to be made” and “my doctor and I selected a treatment option together.” Conversely, the DCS assesses uncertainty experienced by patients regarding their treatment decision (19). When examining the sub-scales of the DCS, while the SDM training program was more effective in two items (“Informed” and “Values”), there were no differences in the other three items (“Support,” “Uncertainty,” and “Effective decision”) between the two groups. This might be because our training program and DAs weighed information sharing with clinicians rather than reducing uncertainties or avoiding decisional regret of patients. It is possible that our training program did not have enough effect for improving patient’ decision conflicts. Therefore, further efforts are needed to improve the contents of the training program and the DAs to focus on the latter aspects. As the baseline differences between the two groups, such as sex and educational background also might have some impact on the results, we need to verify the intervention effects using similar groups. Another possibility of this discrepancy was that we were only able to recruit just over half of the planned participants because of the limitations of the study period. This may have influenced the lack of a significant difference in the DCS. The *p*-value for the DCS was 0.07, indicating a trend toward a significant difference, which could have been achieved if the planned sample size had been met.

Although there are a few SDM training program studies that have reported positive long-term outcomes, we could not find any long-term effects in our findings. Robinson et al. developed the NAVIGATE system for schizophrenia treatment, which included unique elements of detailed first episode-specific psychotropic medication guidelines and a computerized decision support system to facilitate SDM regarding prescriptions (27). They found that the quality of life and psychotic and depressive symptom outcomes were better with those using the system (27). Their study was distinctive as the patient and the physician could discuss the medication regimen in line with the guidelines using the system at each consultation. Thus, our program might need improvements to access the elements of depression guidelines at each consultation. Our training program was only a 1-day program, and adjunctive DA could be a limiting effect for improving patients’ outcomes, especially long-term outcomes. Further booster sessions or regular supervision in a clinical setting might be helpful for better SDM training. For example, an online SDM training program, which is accessible to each clinician, might be useful. Furthermore, we might need to provide opportunities for clinicians and patients to learn the SDM concept and its procedures. Moreover, we should also assess treatment continuation as another long-term outcome in the future.

Despite the aforementioned, the specific feature of this study is providing an opportunity for SDM training for clinicians. The degree of patient involvement in decision-making appears to be influenced by the individual clinician (28). Matthias et al. examined communication between patients and clinicians during psychiatric consultations and found that clinicians tended to initiate most decision-making (29). Even after taking SDM into practice, patients’ preference-based conversations occurred less (30). Accordingly, clinicians should learn principles of patient engagement, train in the SDM approach, and use the appropriate decision-support tools that can promote collaborative deliberation (12). This study is valuable to fulfill this significance. Another feature of this study is adopting decision aids, which were developed incorporating the MDD guidelines with evidence-based recommendations. Sharing evidence with patients is recognized to be crucial, and several treatment guidelines recommend implementing SDM (5, 6). Hoffmann et al. emphasized that even if there is evidence for treatment, without SDM, evidence-based medicine can turn into evidence tyranny (31). Our DAs, which include evidence-based treatment options and the advantages and disadvantages of each option, can play a role in facilitating SDM. This can lead to the implementation of the evidence-based treatments recommended by the guidelines. This is one of the strengths of our study.

However, our study has several limitations. First, we used only self-report questionnaires to measure patients’ outcomes, which might be subject to bias. Second, lacking blinding of participants owing to its randomized design may have affected the self-report questionnaire responses. Third, this study did not evaluate clinicians’ support techniques regarding medical decision-making. Fourth, some control group clinicians may have already performed the SDM approaches. Furthermore, although these clinicians could not use the decision aids, the individuals in this group may have received decision support from clinicians or other healthcare professionals separately. Fifth, some intervention group clinicians may experience the study tasks as burdensome and challenging to incorporate into their routine clinical work. Sixth, we did not evaluate patients’ autonomy level or attitude to decision-making. Some control group patients may already be familiar with participating in medical decision-making. Seventh, this study had a relatively small sample size because of funding limitations, potentially affecting the generalizability of results. Despite these limitations, the use of cluster randomized controlled methods is a strength of this study. Moreover, to the best of our knowledge, this is the first study to develop SDM training for clinicians based on the MDD guidelines; further, this is the first study to investigate this in patients newly diagnosed with MDD to improve perceived participation in decision-making processes.

## Conclusion

In conclusion, a clinician training program based on the Japanese MMD guidelines using related decision aids improved patients’ perceived participation in decision making. Additional research is needed to confirm the efficacy of this training program for the dissemination of SDM as well as the MDD guidelines in clinical settings (32).

## Data availability statement

The raw data supporting the conclusions of this article will be made available by the authors, without undue reservation.

## Ethics statement

The studies involving human participants were reviewed and approved by the Ethics Committee of Kyorin University. The patients/participants provided their written informed consent to participate in this study. Written informed consent was obtained from the individual(s) for the publication of any potentially identifiable images or data included in this article.

## Author contributions

YoT and YA: interpreting the data, drafting the manuscript, and organizing and implementation of this study. YA: drafting the prototypes of decision aids and collecting the data. YA, YoT, TT, and KW: developing the final version of decision aids, organization, and development of a training program and the study design. YoT, YA, TT, YI, and KW: performing the training program. YoT, TT, and KW: recruiting participants. HT: calculating the sample size and deciding the analytic strategy. YuT, MI, and HT: statistical analysis. KW: funding acquisition. All authors revising the manuscript and approved the final version of the manuscript.

## References

[1] James SL Abate D Abate KH Abay SM Abbafati C Abbasi N Global, regional, and national incidence, prevalence, and years lived with disability for 354 diseases and injuries for 195 countries and territories, 1990–2017: A systematic analysis for the global burden of disease study 2017. *Lancet.* (2018) 392:1789–858. 10.1016/S0140-6736(18)32279-7 30496104 PMC6227754

[2] Mathers CD Loncar D. Projections of global mortality and burden of disease from 2002 to 2030. *PLoS Med.* (2006) 3:e442. 10.1371/journal.pmed.0030442 17132052 PMC1664601

[3] Cleare A Pariante CM Young AH Anderson IM Christmas D Cowen PJ Evidence-based guidelines for treating depressive disorders with antidepressants: A revision of the 2008 British association for psychopharmacology guidelines. *J Psychopharmacol.* (2015) 29:459–525. 10.1177/0269881115581093 25969470

[4] Lam RW Kennedy SH Parikh SV MacQueen GM Milev RV Ravindran AV. Canadian network for mood and anxiety treatments (CANMAT) 2016 clinical guidelines for the management of adults with major depressive disorder: Introduction and methods. *Can J Psychiatry.* (2016) 61:506–9. 10.1177/0706743716659061 27486152 PMC4994787

[5] Hasan A Falkai P Wobrock T Lieberman J Glenthoj B Gattaz WF World federation of societies of biological psychiatry (WFSBP) guidelines for biological treatment of schizophrenia, part 2: Update 2012 on the long-term treatment of schizophrenia and management of antipsychotic-induced side effects. *World J Biol Psychiatry.* (2013) 14:2–44. 10.3109/15622975.2012.739708 23216388

[6] Japanese Society of Mood Disorders. *Treatment Guideline II: Major Depressive Disorder.* Tokyo: Igakushoin (2016).

[7] Malhi GS Bell E Bassett D Boyce P Bryant R Hazell P The 2020 royal Australian and New Zealand College of psychiatrists clinical practice guidelines for mood disorders. *Aust N Z J Psychiatry.* (2021) 55:7–117. 10.1177/0004867420979353 33353391

[8] LeBlanc A Herrin J Williams MD Inselman JW Branda ME Shah ND Shared decision making for antidepressants in primary care: A cluster randomized trial. *JAMA Intern Med.* (2015) 175:1761–70. 10.1001/jamainternmed.2015.5214 26414670 PMC4754973

[9] Aoki Y Furuno T Watanabe K Kayama M. Psychiatric outpatients’ experiences with shared decision-making: A qualitative descriptive study. *J Health Commun.* (2019) 2:102–11. 10.2196/34738 35389356 PMC9030980

[10] Perestelo-Perez L Rivero-Santana A Sanchez-Afonso JA Perez-Ramos J Castellano-Fuentes CL Sepucha K Effectiveness of a decision aid for patients with depression: A randomized controlled trial. *Health Expect.* (2017) 20:1096–105. 10.1111/hex.12553 28295915 PMC5600223

[11] Raue PJ Schulberg HC Bruce ML Banerjee S Artis A Espejo M Effectiveness of shared decision-making for elderly depressed minority primary care patients. *Am J Geriatr Psychiatry.* (2019) 27:883–93. 10.1016/j.jagp.2019.02.016 30967321 PMC6646064

[12] Hopwood M. The shared decision-making process in the pharmacological management of depression. *Patient.* (2020) 13:23–30. 10.1007/s40271-019-00383-w 31544218 PMC6957572

[13] American Psychiatric Association. *Diagnostic and Statistical Manual of Mental Disorders.* 5th ed. Arlington, VA: American Psychiatric Association (2013). 10.1176/appi.books.9780890425596

[14] Stacey D Volk RJ. The international patient decision aid standards (IPDAS) collaboration: Evidence update 2.0. *Med Decis Making.* (2021) 41:729–33. 10.1177/0272989X211035681 34416841 PMC8474333

[15] Joseph-Williams N Newcombe R Politi M Durand MA Sivell S Stacey D Toward minimum standards for certifying patient decision aids: A modified delphi consensus process. *Med Decis Making.* (2014) 34:699–710. 10.1177/0272989X13501721 23963501

[16] Aoki Y Takaesu Y Inoue M Furuno T Kobayashi Y Chiba H Seven-day shared decision making for outpatients with first episode of mood disorders among university students: A randomized controlled trial. *Psychiatry Res.* (2019) 281:112531. 10.1016/j.psychres.2019.112531 31521046

[17] Hamann J Watanabe K. Shared decision making in psychiatry. *Japanese J Clin Psychopharmacol.* (2011) 14:678–87.

[18] Kriston L Scholl I Hölzel L Simon D Loh A Härter M. The 9-item shared decision making questionnaire (SDM-Q-9). development and psychometric properties in a primary care sample. *Patient Educ Couns.* (2010) 80:94–9. 10.1016/j.pec.2009.09.034 19879711

[19] O’Connor AM. Validation of a decisional conflict scale. *Med Decis Making.* (1995) 15:25–30. 10.1177/0272989X9501500105 7898294

[20] Nguyen TD Attkisson CC Stegner BL. Assessment of patient satisfaction: Development and refinement of a service evaluation questionnaire. *Eval Program Plann.* (1983) 6:299–313. 10.1016/0149-7189(83)90010-1 10267258

[21] Brooks R. EuroQol: The current state of play. *Health Policy.* (1996) 37:53–72. 10.1016/0168-8510(96)00822-6 10158943

[22] Anderson LA Dedrick RF. Development of the trust in physician scale: A measure to assess interpersonal trust in patient-physician relationships. *Psychol Rep.* (1990) 67:1091–100. 10.2466/pr0.1990.67.3f.1091 2084735

[23] Rush AJ Trivedi MH Ibrahim HM Carmody TJ Arnow B Klein DN The 16-item quick inventory of depressive symptomatology (QIDS), clinician rating (QIDS-C), and self-report (QIDS-SR): A psychometric evaluation in patients with chronic major depression. *Biol Psychiatry.* (2003) 54:573–83. 10.1016/s0006-3223(02)01866-8 12946886

[24] Hamann J Holzhüter F Stecher L Heres S. Shared decision making PLUS – a cluster-randomized trial with inpatients suffering from schizophrenia (SDM-PLUS). *BMC Psychiatry.* (2017) 17:78. 10.1186/s12888-017-1240-3 28231777 PMC5324213

[25] Little RJ Rubin DB. *Statistical Analysis with Missing Data.* (Vol. 793). Hoboken, NJ: John Wiley & Sons (2019). 10.1002/9781119482260

[26] Lipsey MW Wilson DB. The efficacy of psychological, educational, and behavioral treatment. Confirmation from meta-analysis. *Am Psychol.* (1993) 48:1181–209. 10.1037/0003-066X.48.12.1181 8297057

[27] Robinson DG Schooler NR Correll CU John M Kurian BT Marcy P Psychopharmacological treatment in the RAISE-ETP Study: Outcomes of a manual and computer decision support system based intervention. *Am J Psychiatry.* (2018) 175:169–79. 10.1176/appi.ajp.2017.16080919 28945118 PMC5794655

[28] McCabe R Khanom H Bailey P Priebe S. Shared decision-making in ongoing outpatient psychiatric treatment. *Patient Educ Couns.* (2013) 91:326–8. 10.1016/j.pec.2012.12.020 23414657

[29] Matthias MS Salyers MP Rollins AL Frankel RM. Decision making in recovery-oriented mental health care. *Psychiatr Rehabil J.* (2012) 35:305–14. 10.2975/35.4.2012.305.314 22491370 PMC3980461

[30] Fukui S Matthias MS Salyers MP. Core domains of shared decision-making during psychiatric visits: Scientific and preference-based discussions. *Adm Policy Ment Health.* (2015) 42:40–6. 10.1007/s10488-014-0539-3 24500023 PMC4125549

[31] Hoffmann TC Montori VM Del Mar C. The connection between evidence-based medicine and shared decision making. *JAMA.* (2014) 312:1295–6. 10.1001/jama.2014.10186 25268434

[32] Takaesu Y Watanabe K Numata S Iwata M Kudo N Oishi S Improvement of psychiatrists’ clinical knowledge of the treatment guidelines for schizophrenia and major depressive disorders using the ‘effectiveness of guidelines for dissemination and education in psychiatric treatment (EGUIDE)’ project: A nationwide dissemination, education, and evaluation study. *Psychiatry Clin Neurosci.* (2019) 73:642–8. 10.1111/pcn.12911 31437336 PMC6852015

