# Supplementary material for: Implementation of a shared decision-making training program for clinicians based on the major depressive disorder guidelines in Japan: A multi-center cluster randomized trial
Source: Front Psychiatry. 2022 Aug 12;13:967750. doi: 10.3389/fpsyt.2022.967750 (PMC9413755; doi:10.3389/fpsyt.2022.967750)
Supplement: Supplementary file 1 [file Data_Sheet_1.pdf]

● A Decision Aid for Depression ●

治療法を選ぶための手引き

うつ病 ～軽症編～

うつ病の特徴を知って
あなたにあった治療法を一緒に選びましょう

この手引きは**うつ病治療ガイドライン第2版**にもとづいて作成されています

この手引きの使い方 (1)

この手引きは、**軽症うつ病**[1]と診断された方が、医療者と話し合いながら自分にあった治療法を選ぶためのものです。

はじめの診察では、うつ病とその症状の程度、治療の選択肢について、医師と一緒に確認します。そして手引きを持ち帰ってよく読み、つぎの診察で医師と話しあい、治療法を決めます。

手引きをよく読みます
○をつけたりメモ欄に
記入したりします

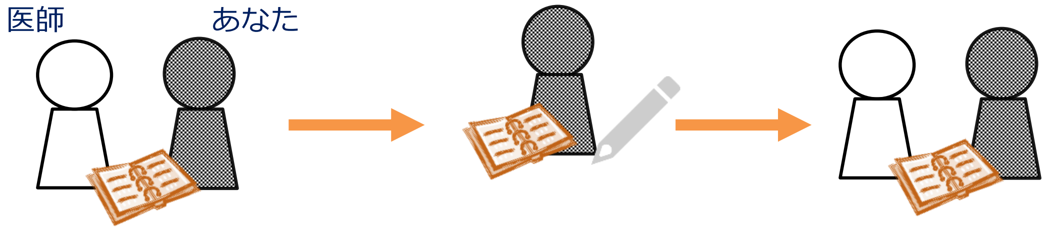

診察で

・うつ病とはどんな状態か
・症状の程度（重症度）
・治療の選択肢
を一緒に確認します

診察で

・あなた：質問する
　医師：回答する
・○をした項目や
　メモした内容について
　話し合います
・治療法を選びます

この手引きの使い方 (2)

うつ病には、症状の軽重があります。

この手引きは、現時点で **軽症うつ病**[1]と診断された方が、うつ病について知り、自分にあった治療法を検討するための手引きです。

まず、前半で「うつ病」について理解します。

そして、後半で治療法を検討していきます。

5～13ページ
・うつ病とは

→

15～27ページ
・治療法を選ぶ
～軽症編～

※ なお、うつ病の中等症以上では、推奨される治療法が異なります[2]。中等症以上については、別冊『治療法を選ぶための手引き うつ病 ～中等症・重症編』を作成しています。

ご希望があればお渡しできますのでお声かけください。

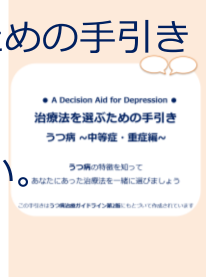

また、現時点では軽症でも、経過のなかで中等症以上になることもあります。その場合は、中等症以上で推奨される治療法を検討します。

もくじ

- うつ病とは……………………………………… 5
- 治療法を選ぶ ～うつ病軽症編～………… 15

うつ病 とは

「うつ病とは何か」を理解しましょう

あてはまるものがありますか？

ここ2週間位の状態として、あてはまるものに✓してみましょう

●抑うつ

- □ 気分の落ち込み
- □ 悲しい気持ち

●興味・喜び

- □ 好きだったことや趣味に取り組めなくなった
- □ 身だしなみ、身の回りのことなどどうでもいい

●食事・体重

- □ 食欲がない または 食べすぎる
- □ 何を口にしても美味しくない
- □ 体重が減った または 増えた

●睡眠

- □ 眠れない または 寝すぎる
- □ 眠りが浅く何度も目が覚めてしまう
- □ 朝早く目が覚めてしまう

1)より

●活動性

□ 体を動かすのがおっくう または 焦って落ち着かない

□ 周りから活動の低下 または 落ち着かなさ を指摘される

●疲労感・気力

□ だるい、疲れやすい

□ 人に会いたくない

●自分や周りについての考え

□ 自分は価値のない人間だと思う

□ 昔の小さなことを思い出しては悩む

□ 周りの人に申し訳ないと思う

●思考力・集中力

□ 集中して取り組むことができない

□ ものごとが決められない

●死について

□ いっそ消えてしまいたい

1)より

うつ病とは

● ただの落ち込みとどう違うのですか？[1]

前頁にあげたものは、うつ病の症状です。人は誰でも辛いことがあれば落ち込みますが、うつ病になると**ほとんど1日中、ほとんど毎日、2週間以上にわたり**、これらの症状に悩まされます。さらに、人間関係や仕事など、日々の生活にも支障をきたすようになります。

この手引きで取り上げている**軽症うつ病**は、日常生活における支障が比較的少ないのが特徴です。

たとえば・・・

「仕事には行っているが同僚とのコミュニケーションがうまくとれなくなった」

「授業に出ているけど前より集中できない」

「誘われれば行くけど友人との交流が減った」

普段の生活は送れていても、本来の自分とは違って、これまで通りにはいかなくなっている状態です。

また軽症うつ病は、下のURL／QRコードより入手できるうつ病チェックリストで、 **6～10**点 の状態とされています[3]。

http://www.mhlw.go.jp/bunya/shougaihoken/kokoro/dl/02.pdf

うつ病とは

●きっかけがあるのでしょうか？[2]

　ストレスがきっかけとなることがあります。ストレスの感じ方は人それぞれで、自分では気付かないこともあります。喜ばしい出来事もストレスになり得ます。

こんなことはありませんでしたか？

☐ 環境の変化

進学、就職、職場の異動、結婚、引っ越し、昇進

☐ 失う体験

家族や親しい友人との別れ、失恋、離婚

☐ 体調の変化

病気、けが

●自分だけでしょうか？

　日本人100人のうち３～7人が一生に一度はうつ病になるとされています[4]。また、働き盛りの**30～50**歳代に多いのも特徴で[4]、決して珍しいことではありません。

うつ病とは

●なぜ起こるのでしょうか？[2)]

　抱えきれないストレスに直面した際、周囲のサポートや睡眠が十分に得られないと、脳が状況を処理できず、ものの見方が否定的になります。すると、前にも増して周囲のサポートがないと思え、小さなことでもストレスに思えてきます。こうして、**ぐるぐると悪循環が形成され、抜け出せなくなっている**のがうつ病です。
決して「気持ちが弱いから」起こるのではありません。

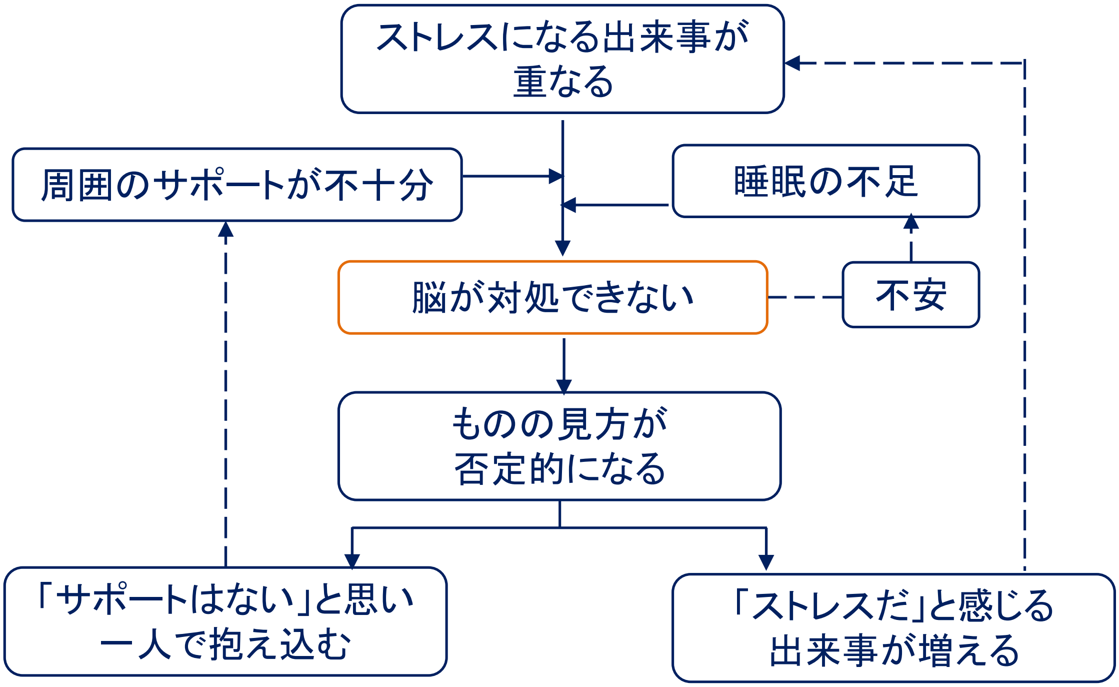

図1.【うつ病が起こる悪循環の仕組み】2)より引用

うつ病とは

●思うようにいかないのはなぜですか？

　うつ病はこころのエネルギー切れと言えます。ガソリンの切れた車のような状態ですから、周りにいくら励まされても、思うように前に進めないのは当然なのです。家族や周りの人もこの状態を理解することが大切です。また、適切な判断もできなくなっています。結婚、転退職、財産の処分など、人生の大きな決断は、回復を待ってから行うようにしましょう[2)]。

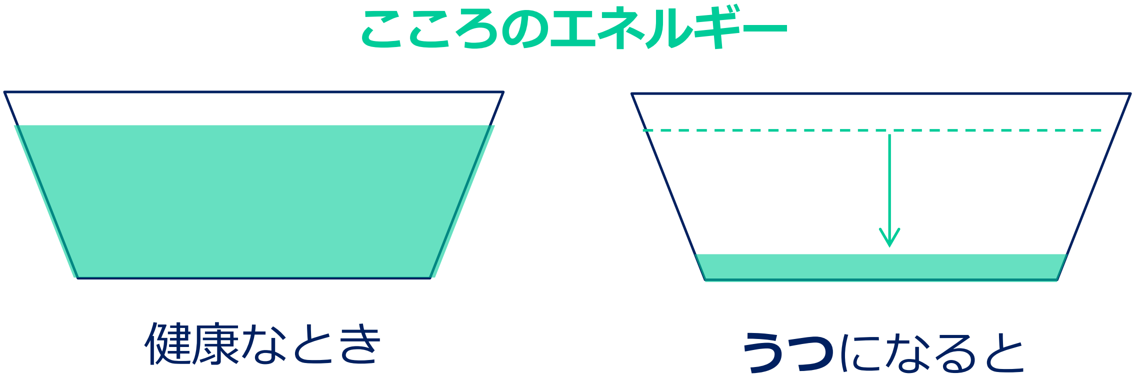

うつ病とは

●自分でできることはありますか？[2)]

　規則正しい生活が基本です。

・夜間に十分な睡眠をとり、朝は外光を浴びましょう
・飲酒は睡眠の質を下げるため控えます
・バランスのよい食事を心がけます
・適度に体を動かし、リラックスできる時間を作ります
・周囲にサポートを求め、人の力も借りましょう
・物事に優先順位をつけ、何事も8割達成を目指します

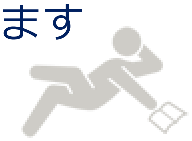

　睡眠時間や日々の活動を記録することもお勧めです。また、その日の気分に点数をつけてみましょう。自分の生活が客観的に把握できるようになります。

日記のように・・・

	(午前) 0	2	4	6	8	10	(午後) 0	2	4	6	8	10	12	気分 -2	-1	0	1	2	日常行動
1日 ( )																✔			頭痛あり
2日 ( )															✔				仕事を休んだ
3日 ( )																✔			友人と出かけた
4日 ( )																			
5日 ( )																			
6日 ( )																			

睡眠・覚醒リズム表はこちらからダウンロードできます（日本うつ病学会ウェブサイト）
http://www.secretariat.ne.jp/jsmd/sokyoku/pdf/suimin_kakusei_rhythm.pdf

うつ病とは

●治りますか？[2]

　うつ病は治ります。ただ、一旦よくなっても再燃することが多いのです。規則正しい生活や周囲のサポート、後述する治療などを組み合わせ、長い目でみながら一緒に取り組んでいきましょう。

　うつ病について、疑問や確認したいことを書いておきましょう。診察で話し合います。

☞ つぎは、**治療法**をみていきます

治療法を選ぶ ～うつ病軽症編～

治療法について医師と話し合う準備をします

①うつ病治療とは

うつ病が起こる悪循環の仕組み（10頁）を形成している要素に働きかけ、この悪循環を断ち切ることが、うつ病の治療です。悪循環を断ち切る方法を　　　　　で示します。

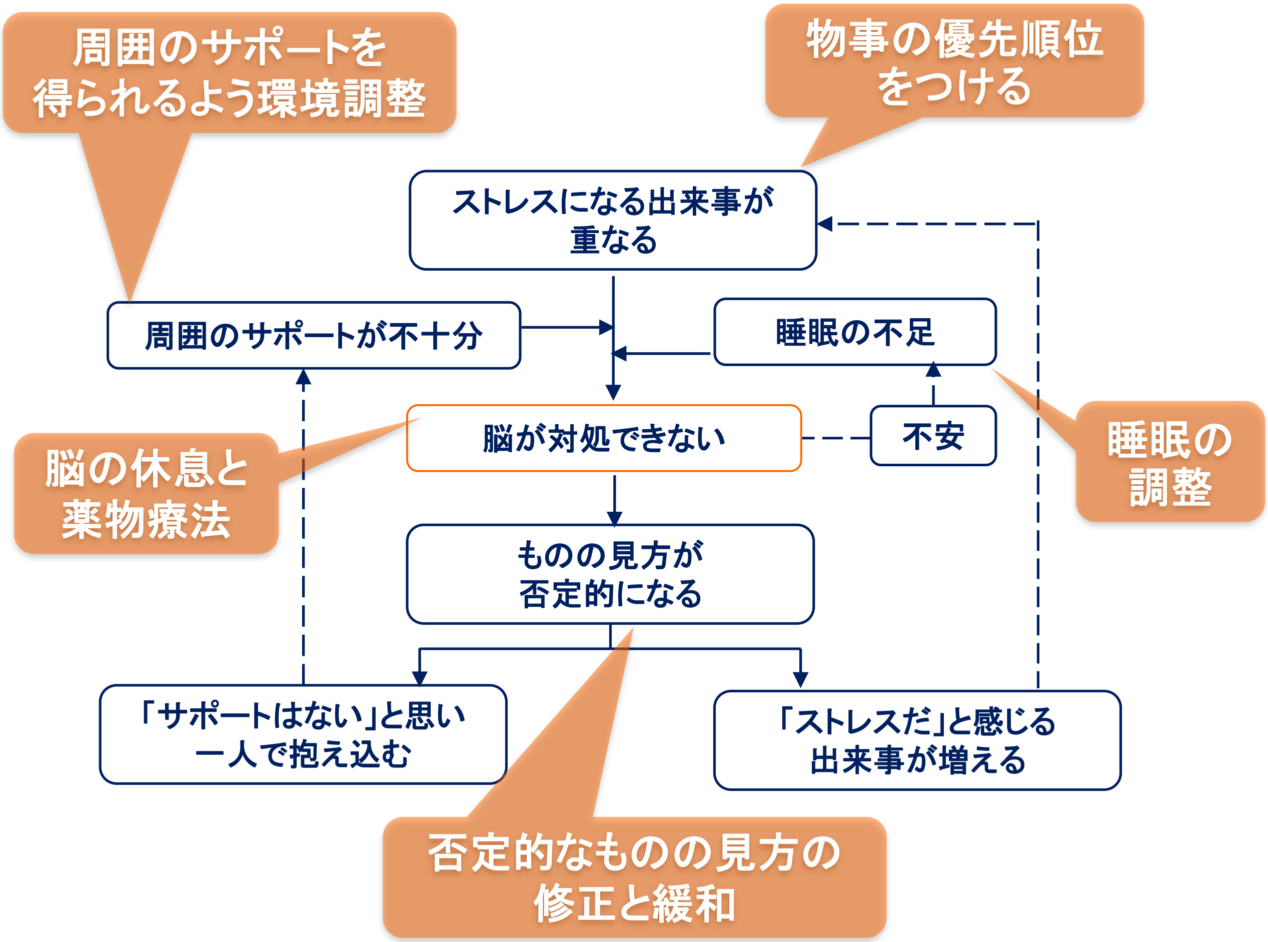

図2.【うつ病の悪循環を断ち切る方法】2)より引用/一部改変

☞ つぎからは、悪循環を断つための具体的な治療法をみていきます

②うつ病の基礎的な治療

うつ病に効果があることがわかっており、うつ病にかかった人すべてに推奨されている治療（うつ病の基礎的な治療）は、『うつ病を理解する』・『医師と話し合う』の2つです。

『うつ病を理解する』

最も大切なのは今の自分の状態を知ることです。この手引きの前半部分「うつ病について」をよく読み、うつ病を理解します。さらに、生活を振り返り、優先順位をつける、周囲に協力を求める等、自分でできることに取り組みます。

『医師と話し合う』

定期的に通院を続け、医師と会い、日々の出来事や困り事について話し合います。

軽症うつ病の場合、この基礎的な治療だけでもうつが改善することがわかっています。そして、これに加える選択治療として、薬物療法と体系化された精神療法があります。

☞つぎに、薬物療法と体系化された精神療法をみていきます

2)より

③基礎的な治療に加える選択肢を知る

選択肢❶ 薬物療法

　抗うつ薬による治療です。
セロトニンなどの脳の神経伝達物質のアンバランスを整え、脳の休息を促します。睡眠の調整にも働きます。

選択肢❷ 体系化された精神療法

　訓練を受けた専門家（医師や心理士、看護師）のもとで行います。
例）認知行動療法：否定的な認知の修正と緩和を目的とした治療です。感情や行動に影響を及ぼしている偏った物事の捉え方を、現実的で幅広い捉え方に修正していきます。

　基礎的な治療に加え、これらを単独で、あるいは併せて選択することも可能です。ただし、それぞれの治療法には利点がある一方で欠点もあります。特徴をよく見比べ、あなたにあった治療法を一緒に選びましょう。

☞ 次頁で特徴を比較してみます

2)より

④加える治療の選択肢を比較する

	❶ 薬物療法	❷ 体系化された精神療法<br>例. 認知行動療法[2]
利点 ☺	うつが軽快する（ただし偽薬と効果の差はないという報告もある）[2]	うつが軽快する
効果が出るまでの時間	2~4週間[5]	多くは16週程度の治療コースの終了後
治療に要する時間	・2～3週毎に通院する<br>・再発予防には回復後も半年は続けることが推奨されている[6]	原則、毎週1回30分以上の面接を16回行う（個々で異なる）
欠点 ☹	・嘔気や嘔吐、下痢など何らかの副作用を経験する場合が多い<br>（副作用の詳細は25頁）<br>・急に止めると頭痛やめまいなどの中断症状が出ることがある<br>（詳しくは25頁）	・実施している施設が限られる<br>・実施する治療者の技術に差がある可能性がある<br>・自身の事や気持ちを話すことは難しいと感じるかもしれない

⑤情報を整理する

ここでは、うつ病治療に関するその他の情報を整理しておきます

● **『仕事（学校）を休んだ方がよいのでしょうか？』**

軽症うつ病では、休養よりも規則正しい生活を送ることを優先したほうがよい場合もあります。仕事（学校）の内容も含め、今の仕事（学校）を休むことのあなたにとっての利点・欠点を医師とよく話し合い決めます。

● **『体を動かしたほうがいいのでしょうか？』**

運動のうつ病への効果ははっきりはわかっていません。自己流の激しい運動では、反対に心身の調子を損ねる恐れがあります。心地よい程度に体を動かせるよう医師と相談しながら行います。

● **『食事やサプリメントはどうでしょうか？』**

様々な検証が行われていますが、どれも科学的根拠は乏しいのが現状です。バランスのよい食事をこころがけましょう。

● **『できれば薬は使いたくないのですが・・・』**

軽症うつ病の場合、先述の「基礎的な治療」を行うだけでも、うつが改善することがわかっています。ただし、症状が重くなってきた場合や、なかなかよくならない場合は、薬や体系化された精神療法を追加することが推奨されます。

2)より

⑤情報を整理する

● **『薬は飲み始めると止められなくなるのでは？』**

抗うつ薬には、耐性や依存性はありません。一定期間飲み続け、少しずつ減らし、薬なしでも生活できるよう目指します。

● **『薬で性格が変わってしまうのでは？』**

効果がでてくると、感じ方や考え方が前向きになっていきますが、薬で性格まで変わることはありません。

● **『妊娠・授乳中でも薬を飲んで大丈夫でしょうか？』**

まだ裏付けが十分ではありませんが、妊娠・授乳中の抗うつ薬の服用による胎児への悪影響が報告されています[7)]。 妊娠の可能性がある場合は、医師とよく話し合います。

軽症うつ病の治療について理解できましたか？
☞ 次頁で、治療に関するあなたの希望を整理しましょう

⑥治療に関する希望を整理する

●考えを整理しましょう

あなたにとって、治療に関する以下の内容は、
どのくらい重要ですか？**0～5**で重みづけをしてみましょう。

内容	重要でない					重要である
うつの症状が軽快する	0	1	2	3	4	5
生活や仕事がしやすい	0	1	2	3	4	5
治療に要する時間	0	1	2	3	4	5
効果が現れるまでの時間	0	1	2	3	4	5
副作用に関すること	0	1	2	3	4	5
その他（気になること）						
・	0	1	2	3	4	5
・	0	1	2	3	4	5
・	0	1	2	3	4	5

☞ つぎに、薬を希望する場合の薬の選択肢をみていきます

–薬を希望する方へ-

⑦薬の選択肢を知る [2,8,9]

　薬を希望する場合、薬には複数の選択肢があります。効き目に明らかな優劣の差はないとされ[8]、薬ごとの特徴を比較し、あなたの好みにあった薬を選択します。

	薬のなまえ	飲み方	形	運転 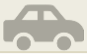	後発薬※
SSRI	フルボキサミン	1日<br>1～4回	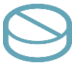	✕<br>禁止	○
	パロキセチン	1日1回<br>夕食後	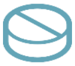	！<br>注意	○
	セルトラリン	1日1回	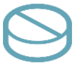	！<br>注意	○
	エスシタロプラム	1日1回<br>夕食後	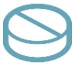	！<br>注意	
SNRI	デュロキセチン	1日<br>1～3回食後	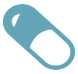	！<br>注意	
	ベンラファキシン	1日1回<br>朝食後	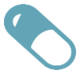	！<br>注意	
	ミルナシプラン	1日1回<br>食後	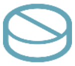	！<br>注意	○
NaSSA	ミルタザピン	1日1回<br>寝る前	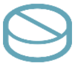	✕<br>禁止	

※後発薬…新薬の特許が切れたあとに他の製薬会社が同じ有効成分で製造・供給する薬。先発品に比べて安価。

⑧薬の選択肢を比較する -効果と副作用の比較-[9,10]

	薬のなまえ	効果	便秘口渇	吐き気下痢	眠気過鎮静	不眠焦燥	性欲減退	ふらつき	体重増加
SSRI	フルボキサミン	不安を和らげる 強迫・過食にも効く	●	●●●		●	●		
	パロキセチン		●	●●		●●	●●		●
	セルトラリン			●●		●●	●●		
	エスシタロプラム			●●		●●	●●		
SNRI	デュロキセチン	意欲を出す		●●		●●	●		
	ベンラファキシン			●●		●●	●●		
	ミルナシプラン			●●		●●	●●		
NaSSA	ミルタザピン	早く効く			●●			●	●●

【注意】

・どの抗うつ薬でも、ときにイライラし衝動性が高まることがあります（とくに24歳以下の若年）。その場合すぐに飲むのをやめてください。

・どの抗うつ薬でも、急に飲むのを止めると、頭痛、めまい、吐き気などの中断症状がでることがあります。薬は状態がよくなったら少しずつ減らします。

・エスシタロプラムでは不整脈が現れる可能性があり、もともと不整脈のある方は服用できません。

⑨薬に関する希望を整理する

●考えを整理しましょう

あなたにとって、以下の薬の特徴は、どのくらい重要ですか？
0～5で重みづけをしてみましょう。

内容	重要でない					重要である
不安を和らげる	0	1	2	3	4	5
意欲も出す	0	1	2	3	4	5
効き始めるのが早い	0	1	2	3	4	5
便秘・口渇	0	1	2	3	4	5
消化器への影響	0	1	2	3	4	5
睡眠への影響	0	1	2	3	4	5
性機能への影響	0	1	2	3	4	5
体重への影響	0	1	2	3	4	5
不眠・焦りの出現	0	1	2	3	4	5
中断による症状	0	1	2	3	4	5
その他（気になること）						
・	0	1	2	3	4	5
・	0	1	2	3	4	5
・	0	1	2	3	4	5

⑩話し合う準備をする

あなたの重みづけや考えをもとに、**治療や薬について医師と話し合います**。

考えたことや疑問を書いておきましょう

おわりに

●自分にあった対処法・治療法を選ぶために

　治療の選択肢には、それぞれ利点と欠点があります。この手引きは、それらをよく理解し、自分にとって重要なことを明らかにしながら医療者と話し合い、あなたに合った選択ができるよう作られています。

●手引きの開発プロセス

　この手引きに掲載した情報は、うつ病治療ガイドライン第2版にもとづいています。さらに、うつ病の治療を経験されたことのある方々の声や意見を反映させました。精神科の専門家のチェックも受けています。なお、企業などからの資金援助は受けていません。

●手引きの更新

　この手引きは、必要に応じて見直しと更新をおこないます。

※ ここに掲載された情報は、医療者と話し合いながら対処法や治療法を決める際の手引きとなるものであり、医療者のアドバイスの代わりになるものではありません。

引用・参考文献

1) 米精神医学会. 精神疾患の診断・統計マニュアル DSM-5. 医学書院, 2014.

2) 日本うつ病学会. うつ病治療ガイドライン第2版. 医学書院, 2017.

3) 簡易抑うつ症状尺度QIDS -J. 厚生労働省ウェブサイト,
available from: http://www.mhlw.go.jp/bunya/shougaihoken/kokoro/dl/02.pdf

4) 川上憲人. 医学のあゆみ, 219(13), 925-929, 2006.

5) Perraton LG., et al. J Eval Clin Pract, 16(3), 597-604, 2010.

6) Reimherr FW., et al. Am J Psychiatry, 155(9), 1247-1253, 1998.

7) Udechuku A., et al. Aust N Z J Psychiatry, 44(11), 978-996, 2010.

8) Grtlehner G., et al. Ann Intern Med, 155(11), 772-785, 2011.

9) 渡邉衡一郎.神経系に作用する薬剤 In 今日の治療薬 2018. 南江堂, 2018.

10) Bauer M., et al. World J Biol Psychiatry, 14(5), 334-385, 2013.

作成者・青木裕見 聖路加国際大学大学院看護学研究科

・坪井貴嗣 杏林大学医学部精神神経科学教室

・高江洲義和 杏林大学医学部精神神経科学教室

・渡邉衡一郎 杏林大学医学部精神神経科学教室

作成日：2018年8月2日　　更新予定日：2019年8月

この手引きは、国立研究開発法人日本医療研究開発機構による

助成により作成されたものです。
