# Supplementary material for: Implementation of a shared decision-making training program for clinicians based on the major depressive disorder guidelines in Japan: A multi-center cluster randomized trial
Source: Front Psychiatry. 2022 Aug 12;13:967750. doi: 10.3389/fpsyt.2022.967750 (PMC9413755; doi:10.3389/fpsyt.2022.967750)
Supplement: Supplementary file 2 [file Data_Sheet_2.pdf]

● A Decision Aid for Depression ●

治療法を選ぶための手引き

うつ病 ～中等症・重症編～

うつ病の特徴を知って
あなたにあった治療法を一緒に選びましょう

この手引きは**うつ病治療ガイドライン第2版**にもとづいて作成されています

この手引きの使い方 (1)

この手引きは、**中等症や重症のうつ病**[1)]と診断された方が、医療者と話し合いながら自分にあった治療法を選ぶためのものです。

はじめの診察では、うつ病とその症状の程度、治療の選択肢について、医師と一緒に確認します。そして手引きを持ち帰ってよく読み、つぎの診察で医師と話しあい、治療法を決めます。

手引きをよく読みます
○をつけたりメモ欄に
記入したりします

無理せず、体調と
相談しながらできる
ところまで行います

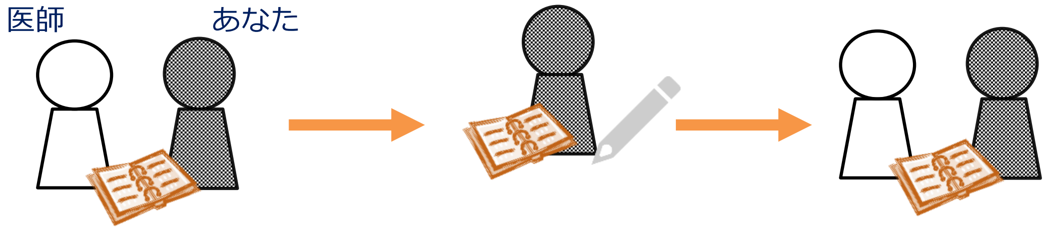

診察で
・うつ病とはどんな状態か
・症状の程度（重症度）
・治療の選択肢
を一緒に確認します

診察で
・あなた：質問する
　医師：回答する
・○をした項目や
　メモした内容について
　話し合います
・治療法を選びます

この手引きの使い方 (2)

　うつ病には、症状の軽重があります。この手引きは、**中等症や重症のうつ病**[1)]と診断された方が、うつ病について知り、自分にあった治療を検討するための手引きです。

まず、前半で「うつ病」について理解します。
そして、後半で治療法を検討していきます。

5～13ページ
・うつ病とは

→

15～27ページ
・治療法を選ぶ
～中等症・重症編～

※ なお、うつ病は症状の軽重によって推奨される治療法が異なります[2)]。軽症のうつ病については、別冊『治療法を選ぶための手引き うつ病 ～軽症編』を作成しています。
ご希望があればお渡しできますのでお声かけください。

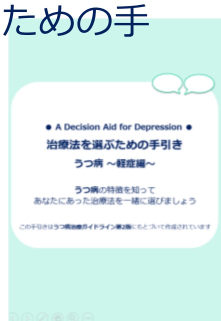

ただし、症状が軽くても、再発のうつ病やなかなかよくならないうつ病では、この手引きでお伝えする中等症・重症の治療法を検討することが推奨されています[2)]。

もくじ

- うつ病とは………………………………… 5

- 治療法を選ぶ ～うつ病中等症・重症編～… 15

うつ病 とは

「うつ病とは何か」を理解しましょう

あてはまるものがありますか？

ここ2週間位の状態として、あてはまるものに✓してみましょう

●抑うつ

- □ 気分の落ち込み
- □ 悲しい気持ち

●興味・喜び

- □ 好きだったことや趣味に取り組めなくなった
- □ 身だしなみ、身の回りのことなどどうでもいい

●食事・体重

- □ 食欲がない または 食べすぎる
- □ 何を口にしても美味しくない
- □ 体重が減った または 増えた

●睡眠

- □ 眠れない または 寝すぎる
- □ 眠りが浅く何度も目が覚めてしまう
- □ 朝早く目が覚めてしまう

1)より

● **活動性**

□ 体を動かすのがおっくう または 焦って落ち着かない

□ 周りから活動の低下 または 落ち着かなさ を指摘される

● **疲労感・気力**

□ だるい、疲れやすい

□ 人に会いたくない

● **自分や周りについての考え**

□ 自分は価値のない人間である

□ 昔の小さなことを思い出しては悩む

□ 周りの人に申し訳ない

● **思考力・集中力**

□ 集中して取り組むことができない

□ ものごとが決められない

● **死について**

□ いっそ消えてしまいたい

1)より

うつ病とは

● どのような状態なのでしょうか？[1]

　前頁にあげたものは、うつ病の症状です。人は誰でも辛いことがあれば落ち込みますが、うつ病になると**ほとんど1日中、ほとんど毎日、2週間以上にわたり**、これらの症状に悩まされます。さらに、人間関係や仕事など、日々の生活にも支障をきたすようになります。

　この手引きで取り上げている**中等症以上のうつ病**は、日常生活における支障が大きいのが特徴です。

たとえば・・・
「人との交流が乏しくなり自宅にこもるようになった」
「仕事や学校に行けなくなった」
「身の周りのことや家事ができなくなり生活が乱れてきた」

普段通りの生活が送れなくなっている状態と言えます。

また、うつ病の中等症は、下のURL／QRコードより入手できる うつ病チェックリストで **11～15**点、重症は **16**点以上 の状態とされています[3]。

http://www.mhlw.go.jp/bunya/shougaihoken/kokoro/dl/02.pdf

うつ病とは

●きっかけがあるのでしょうか？[2]

ストレスがきっかけとなることがあります。ストレスの感じ方は人それぞれで、自分では気付かないこともあります。喜ばしい出来事もストレスになり得ます。

こんなことはありませんでしたか？

□ 環境の変化

進学、就職、職場の異動、結婚、引っ越し、昇進

□ 失う体験

家族や親しい友人との別れ、失恋、離婚

□ 体調の変化

病気、けが

●自分だけでしょうか？

日本人100人のうち３~7人が一生に一度はうつ病になるとされています[4]。また、働き盛りの**30～50**歳代に多いのも特徴で[4]、決して珍しいことではありません。

うつ病とは

●なぜ起こるのでしょうか？ 2)

抱えきれないストレスに直面した際、周囲のサポートや睡眠が十分に得られないと、脳が状況を処理できず、ものの見方が否定的になります。すると、前にも増して周囲のサポートがないと思え、小さなことでもストレスに思えてきます。こうして、**ぐるぐると悪循環が形成され、抜け出せなくなっている**のがうつ病です。

決して「気持ちが弱いから」起こるのではありません。

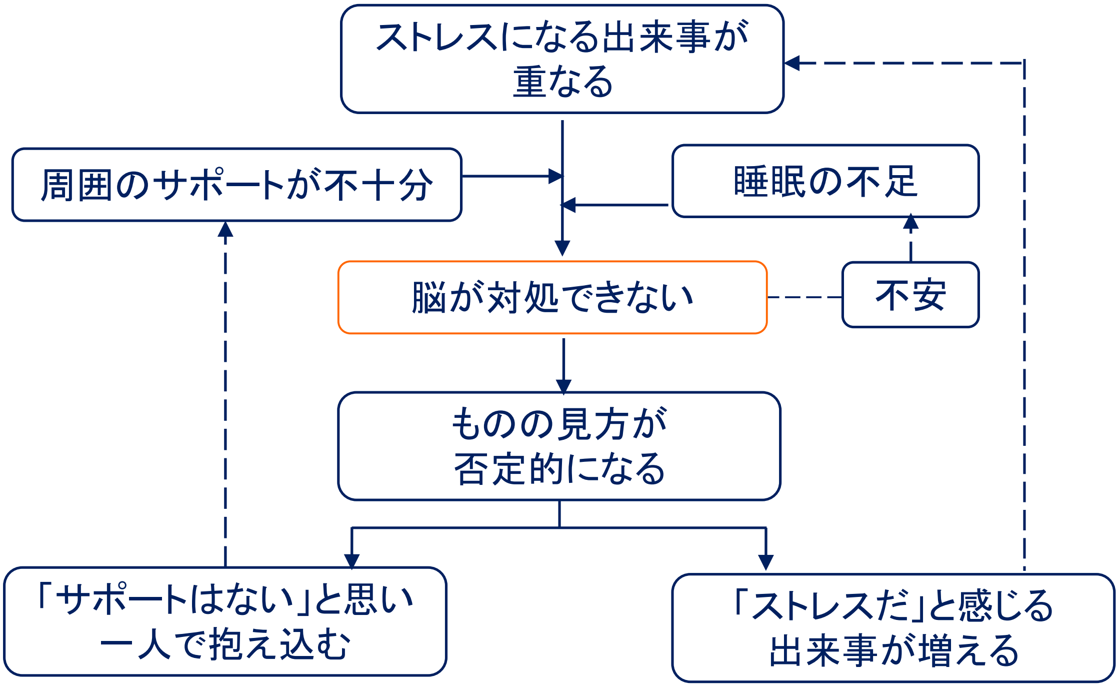

図1.【うつ病が起こる悪循環の仕組み】 2)より引用

うつ病とは

●思うようにいかないのはなぜですか？

うつ病はこころのエネルギー切れと言えます。ガソリンの切れた車のような状態ですから、周りにいくら励まされても、思うように前に進めないのは当然なのです。家族や周りの人もこの状態を理解することが大切です。また、適切な判断もできなくなっています。結婚、転退職、財産の処分など、人生の大きな決断は、回復を待ってから行うようにしましょう[2)]。

こころのエネルギー

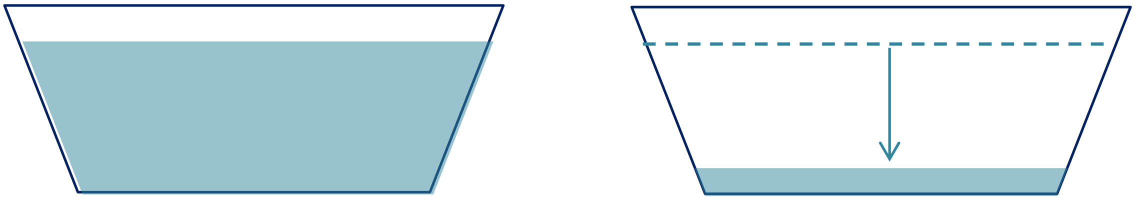

健康なとき　　うつになると

うつ病とは

●自分でできることはありますか？[2)]

規則正しい生活が基本です。

- 夜間に十分な睡眠をとり、朝は外光を浴びましょう
- 飲酒は睡眠の質を下げるため控えます
- バランスのよい食事を心がけます
- 適度に体を動かし、リラックスできる時間をもちます
- 周囲にサポートを求め、人の力も借りましょう
- 物事に優先順位をつけ、何事も8割達成を目指します

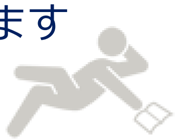

睡眠時間や日々の活動を記録することもお勧めです。また、その日の気分に点数をつけてみましょう。自分の生活が客観的に把握できるようになります。

日記のように・・・

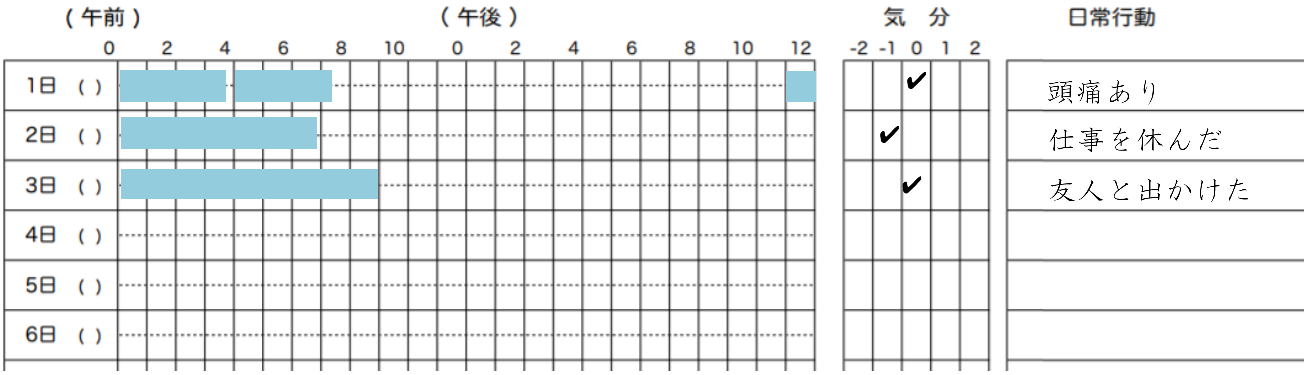

睡眠・覚醒リズム表はこちらからダウンロードできます（日本うつ病学会ウェブサイト）

http://www.secretariat.ne.jp/jsmd/sokyoku/pdf/suimin_kakusei_rhythm.pdf

うつ病とは

●治りますか？[2]

うつ病は治ります。ただ、一旦よくなっても再燃することが多いのです。規則正しい生活や周囲のサポート、後述する治療などを組み合わせ、長い目でみながら一緒に取り組んでいきましょう。

うつ病について、疑問や確認したいことを書いておきましょう。診察で話し合います。

☞ つぎは、**治療法**をみていきます

治療法を選ぶ ～中等症・重症編～

治療法について医師と話し合う準備をします

①うつ病治療とは

うつ病が起こる悪循環の仕組み（10頁）を形成している要素に働きかけ、この悪循環を断ち切ることが、うつ病の治療です。悪循環を断ち切る方法を　　　　で示します。

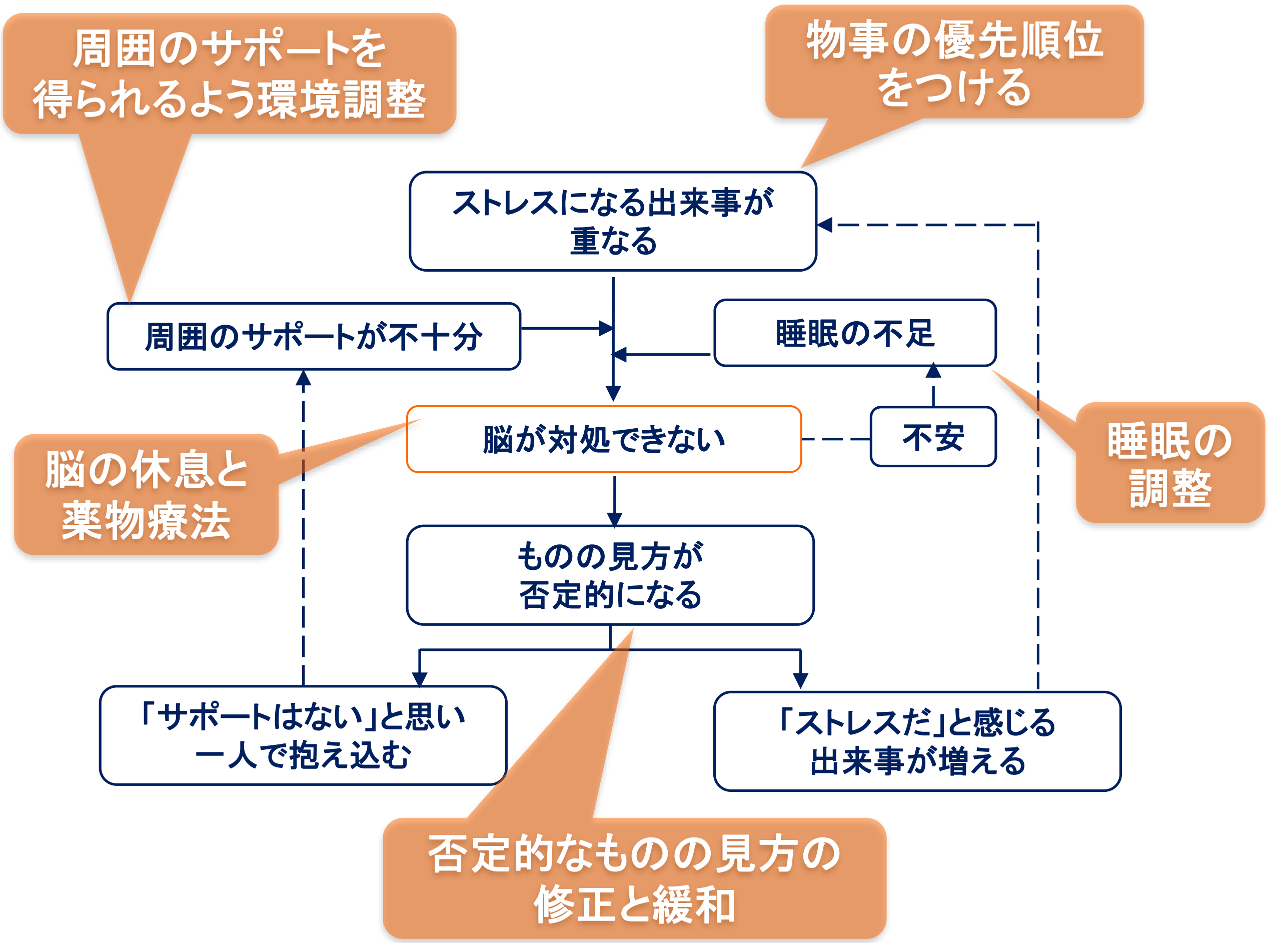

図2.【うつ病の悪循環を断ち切る方法】2)より引用/一部改変

☞ つぎからは、悪循環を断つための具体的な治療法をみていきます

②うつ病の基礎的な治療

うつ病に効果があることがわかっており、うつ病にかかった人すべてに推奨されている治療（うつ病の基礎的な治療）は『うつ病を理解する』・『医師と話し合う』の2つです。

『うつ病を理解する』

最も大切なのは今の自分の状態を知ることです。この手引きの前半部分「うつ病について」をよく読み、うつ病を理解します。さらに、生活を振り返り、優先順位をつける、周囲に協力を求める等、自分でできることに取り組みます。

『医師と話し合う』

定期的に通院を続け、医師と会い、日々の出来事や困り事について話し合います。

中等症以上のうつ病では、この基礎的な治療を行いながら、加えて、薬物療法か修正型電気けいれん療法、あるいはその両方を実施することで、うつが改善することがわかっています。

☞つぎに、薬物療法と修正型電気けいれん療法をみていきます

2)より

③基礎的な治療に加える選択肢を知る

選択肢❶ 薬物療法

　抗うつ薬による治療です。
セロトニンなどの脳の神経伝達物質のアンバランスを整え、脳の休息を促します。睡眠の調整にも働きます。

選択肢❷ 修正型電気けいれん療法

　全身麻酔をして行う治療です。頭部に通電することでけいれんと同様の電気活動を誘発し、うつの症状の改善をはかります。

✓ 体系化された精神療法

　上の選択肢に加えてさらに検討できる治療法として、訓練を受けた専門家（医師や心理士、看護師）のもとで行う体系化された精神療法があります。
例）認知行動療法：否定的な認知の修正と緩和を目的とした治療です。感情や行動に影響を及ぼしている偏った物事の捉え方を、現実的で幅広い捉え方に修正していきます。

2)より

それぞれの治療法には、利点がある一方で欠点もあります。特徴をよく見比べて、あなたにあった治療法を一緒にみつけていきましょう。

☞ 次頁で特徴を比較してみます

④加える治療の選択肢を比較する

	❶ 薬物療法	❷ 修正型電気けいれん療法
利点	うつが改善する[5,6]	うつが改善する[9]<br>自殺抑制の効果がある[10]
効果が出るまでの時間	飲み始めて2~4週間[7]	実施後、すぐに効果がでる[11]
治療に要する時間	・2～3週毎に通院する<br><br>・再発予防には回復後も半年は続けることが推奨されている[8]	・入院して検査等を行ってから実施するため、開始までに数日を要する
欠点	・嘔気や嘔吐、下痢など何らかの副作用を経験する場合が多い（詳しくは24・25頁）<br><br>・急に止めると頭痛やめまいなどの中断症状が出ることがある（詳しくは24・25頁）	・実施後に副作用（頭痛、筋肉痛、通電後の一過性の高血圧・せん妄・記憶障害など)を経験することがある[2]<br><br>・実施後によくなっても、再燃する可能性がある[2]<br><br>・脳や心血管の病気があると実施できない[2]

❶薬・❷修正型電気けいれん療法 に追加できる治療法

	**体系化された精神療法**<br>**例. 認知行動療法** [2]
**利点**	うつが改善する
**効果が出るまでの時間**	多くは16週程度の治療コースの終了後
**治療に要する時間**	原則、毎週1回30分以上の面接を<br>16回行う（個々で異なる）
**欠点**	・実施している施設が限られる<br>・実施する治療者の技術に差がある<br>可能性がある<br>・自身の事や気持ちを話すことは<br>難しいと感じるかもしれない

⑤情報を整理する

ここでは、うつ病治療に関するその他の情報を整理しておきます

● **『薬は飲み始めると止められなくなるのでは？』**

うつ病治療に使われる抗うつ薬には耐性や依存性はありません。

● **『薬づけになりそうで心配です・・・』**

薬は原則、抗うつ薬のうちいずれか1種類を使用し、2種類以上を一緒に飲むことは推奨されません。1つの抗うつ薬でよくならないときは、他の抗うつ薬に変更したり、抗うつ薬以外の薬を補助剤として加えたりすることがあります。ただし、その場合も最小限とし、多数の併用は推奨されません。2)より

● **『妊娠・授乳中でも薬を飲んで大丈夫でしょうか？』**

まだ裏付けが十分ではありませんが、妊娠・授乳中の抗うつ薬の服用による胎児への悪影響が報告されています[12)]。 妊娠の可能性がある場合は、医師とよく話し合います。

● **『うつ病には認知行動療法がよいと聞いたのですが』**

認知行動療法には再発予防の効果があるとされています[2)]。ただし、中等症以上のうつ病では、認知行動療法のみを行うことは推奨されず、薬や修正型電気けいれん療法の追加治療として検討されます[2)]。導入に際しては 医師とよく話し合い決めます。

☞ 次頁で、治療に関するあなたの希望を整理しましょう

⑥治療に関する希望を整理する

●考えを整理しましょう

あなたにとって、うつ病の治療に関する以下の内容は、どのくらい重要ですか？**0～5**で重みづけをしてみましょう。

内容	重要でない					重要である
うつの症状が改善する	0	1	2	3	4	5
仕事や生活がしやすい	0	1	2	3	4	5
治療に要する時間	0	1	2	3	4	5
効果が現れるまでの時間	0	1	2	3	4	5
副作用に関すること	0	1	2	3	4	5
症状の再燃の可能性	0	1	2	3	4	5
その他（気になること）						
・	0	1	2	3	4	5
・	0	1	2	3	4	5
・	0	1	2	3	4	5
・	0	1	2	3	4	5

☞ つぎに、薬を希望する場合の薬の選択肢をみていきます

-薬を希望する場合-

⑧薬の選択肢を比較する -効果と副作用の比較- [2,13,14]

抗うつ薬は複数あります。薬ごとの特徴を比較し、あなたの好みにあった薬を選択します。

	薬のなまえ	😊	便秘 口渇	吐き気 下痢	眠気 過鎮静	不眠 焦燥	性欲 減退	ふらつき	体重 増加
SSRI	フルボキサミン	不安に効く	●	●●●		●	●		
	パロキセチン		●	●●		●●	●●		●
	セルトラリン			●●		●●	●●		
	エスシタロプラム			●●		●●	●●		
SNRI	デュロキセチン	意欲を出す		●●		●●	●		
	ベンラファキシン			●●		●●	●●		
	ミルナシプラン			●●		●●	●●		
NaSSA	ミルタザピン	早く効く			●●			●	●●

	薬のなまえ		便秘<br>口渇	吐き気<br>下痢	眠気<br>過鎮静	不眠<br>焦燥	性欲<br>減退	ふら<br>つき	体重<br>増加
TCA/non-TCA	ミアンセリン	うつが重い場合により効果がある	●		●●			●	●
	マプロチリン		●●		●●		●	●●	●●
	アミトリプチリン		●●●		●●●		●	●●●	●●●
	イミプラミン		●●		●	●●	●	●●	●●
	クロミプラミン		●●●	●	●	●	●●	●●	●●
	ノルトリプチリン		●		●	●	●	●	●
	アモキサピン		●●●		●	●●	●	●	●

【注意】

・どの抗うつ薬でも、ときにイライラし衝動性が高まることがあります（とくに24歳以下の若年）。その場合すぐに飲むのをやめてください。

・どの抗うつ薬でも、急に飲むのを止めると、頭痛、めまい、吐き気などの中断症状がでることがあります。薬は状態がよくなったら少しずつ減らします。

・エスシタロプラム、アミトリプチンでは不整脈が生じる可能性があります（エスシタロプラムはもともと不整脈のある方は服用できません）

⑨薬に関する希望を整理する

●考えを整理しましょう

あなたにとって、以下の薬の特徴は、どのくらい重要ですか？ **0～5**で重みづけをしてみましょう。

内容	重要でない					重要である
不安を和らげる	0	1	2	3	4	5
意欲も出す	0	1	2	3	4	5
効き始めるのが早い	0	1	2	3	4	5
便秘・口渇	0	1	2	3	4	5
消化器への影響	0	1	2	3	4	5
睡眠への影響	0	1	2	3	4	5
性機能への影響	0	1	2	3	4	5
体重への影響	0	1	2	3	4	5
不眠・焦りの出現	0	1	2	3	4	5
中断による症状	0	1	2	3	4	5
その他（気になること）						
・	0	1	2	3	4	5
・	0	1	2	3	4	5
・	0	1	2	3	4	5

⑩話し合う準備をする

　あなたの重みづけや考えをもとに、**治療や薬について医師と話し合います**。

考えたことや疑問を書いておきましょう

おわりに

●自分にあった対処法・治療法を選ぶために

　治療の選択肢には、それぞれ利点と欠点があります。この手引きは、それらをよく理解し、自分にとって重要なことを明らかにしながら医療者と話し合い、あなたに合った選択ができるよう作られています。

●手引きの開発プロセス

　この手引きに掲載した情報は、うつ病治療ガイドライン第2版にもとづいています。さらに、うつ病の治療を経験されたことのある方々の声や意見を反映させました。精神科の専門家のチェックも受けています。なお、企業などからの資金援助は受けていません。

●手引きの更新

　この手引きは、必要に応じて見直しと更新をおこないます。

※ ここに掲載された情報は、医療者と話し合いながら対処法や治療法を決める際の手引きとなるものであり、医療者のアドバイスの代わりになるものではありません。

引用・参考文献
1) 米精神医学会. 精神疾患の診断・統計マニュアル DSM-5. 医学書院, 2014.
2) 日本うつ病学会. うつ病治療ガイドライン第2版. 医学書院, 2017.
3) 簡易抑うつ症状尺度QIDS -J. 厚生労働省ウェブサイト,
available from: http://www.mhlw.go.jp/bunya/shougaihoken/kokoro/dl/02.pdf
4) 川上憲人. 医学のあゆみ, 219(13), 925-929, 2006.
5) Arroll B., et al. Cochrane Database Syst Rev, CD007954, 2009.
6) Baghai TC., et al. Eur Arch Psychiatry Clin Neurosci, 262(1), 13-22, 2012.
7) Perraton LG., et al. J Eval Clin Pract, 16(3), 597-604, 2010.
8) Reimherr FW., et al. Am J Psychiatry, 155(9), 1247-1253, 1998.
9) The UK ECT Review Group. Lancet 361(9360), 799-808, 2003.
10)Kellner CH., et al. Am J Psychiatry, 162(5), 977-982, 2005.
11)Husain MM., et al. J Clin Psychiatry , 65(4),485-491, 2004.
12) Udechuku A., et al. Aust N Z J Psychiatry, 44(11), 978-996, 2010.
13) Bauer M., et al. World J Biol Psychiatry, 14(5), 334-385, 2013.
14) 渡邉衡一郎.神経系に作用する薬剤 In 今日の治療薬2018. 南江堂, 2018.

作成者・青木裕見 聖路加国際大学大学院看護学研究科
・坪井貴嗣 杏林大学医学部精神神経科学教室
・高江洲義和 杏林大学医学部精神神経科学教室
・渡邊衡一郎 杏林大学医学部精神神経科学教室

作成日：2018年8月2日　　更新予定日：2019年8月

この手引きは、国立研究開発法人 日本医療研究開発機構による
助成により作成されたものです。
