# Supplementary material for: Implementation of a shared decision-making training program for clinicians based on the major depressive disorder guidelines in Japan: A multi-center cluster randomized trial
Source: Front Psychiatry. 2022 Aug 12;13:967750. doi: 10.3389/fpsyt.2022.967750 (PMC9413755; doi:10.3389/fpsyt.2022.967750)
Supplement: Supplementary file 3 [file Table_1.pdf]

Shared Decision Making and Major Depressive Disorder Guidelines: One-Day Training Program for Clinicians

**10:00**	**Opening remarks**	
10:00-10:10	Outline of training program	Koichiro WATANABE
**10:10-11:30**	**Lectures for MDD practice guidelines**	
10:10-10:40	Treatment planning	K. WATANABE
10:40-11:10	Treatments for mild, moderate, and severe depression	Takashi TSUBOI
11:10-11:30	Sleep disorder in depression	Yoshikazu TAKAESU
**11:30-11:50**	**Guidance for cluster RCT**	Y. TAKAESU
**11:50-13:00**	**Lunch**	
12:00-12:30	Luncheon lecture “Recovery from depression and SDM”	K. WATANABE
**13:00-14:35**	**SDM role playing – general SDM skills**	
13:00-13:20	Lecture “SDM in depression treatment”	Yumi AOKI
13:25-14:05	Small group role playing	
	Facilitators	K. WATANABE, Y. TAKAESU, T. TSUBOI, Y. AOKI
14:15-14:35	Group discussion, presentation, plenary discussion	
**14:35-14:50**	**Tea break**	
**14:50-16:35**	**SDM role playing – focusing on social functioning and recovery**	
14:50-15:00	Lecture “Social functioning and recovery in depression”	Y. TAKAESU
15:05-15:55	Small group role playing	
	Facilitators	K. WATANABE, Y. TAKAESU, T. TSUBOI, Y. AOKI
16:05-16:35	Group discussion, presentation, plenary discussion	
**16:35-**	**Summary of main points**<br>• Feedback<br>• Q&A session	K. WATANABE
**17:00**	**Closing**	
